# Potential Involvement of lncRNAs in the Modulation of the Transcriptome Response to Nodavirus Challenge in European Sea Bass (*Dicentrarchus labrax* L.)

**DOI:** 10.3390/biology9070165

**Published:** 2020-07-15

**Authors:** Patricia Pereiro, Raquel Lama, Rebeca Moreira, Valentina Valenzuela-Muñoz, Cristian Gallardo-Escárate, Beatriz Novoa, Antonio Figueras

**Affiliations:** 1 Instituto de Investigaciones Marinas (IIM), Consejo Superior de Investigaciones Científicas (CSIC), C/Eduardo Cabello 6, 36208 Vigo, Spain; patriciapereiro@iim.csic.es (P.P.); raquell@iim.csic.es (R.L.); rebecamoreira@iim.csic.es (R.M.); beatriznovoa@iim.csic.es (B.N.); 2 Laboratory of Biotechnology and Aquatic Genomics, Interdisciplinary Center for Aquaculture Research (INCAR), University of Concepción, Concepción 4030000, Chile; valevalenzuela@gmail.com (V.V.-M.); crisgallardo@oceanografia.udec.cl (C.G.-E.)

**Keywords:** RNA-Seq, lncRNAs, *Dicentrarchus labrax*, viral infection, nodavirus, immune response

## Abstract

Long noncoding RNAs (lncRNAs) are being increasingly recognised as key modulators of various biological mechanisms, including the immune response. Although investigations in teleosts are still lagging behind those conducted in mammals, current research indicates that lncRNAs play a pivotal role in the response of fish to a variety of pathogens. During the last several years, interest in lncRNAs has increased considerably, and a small but notable number of publications have reported the modulation of the lncRNA profile in some fish species after pathogen challenge. This study was the first to identify lncRNAs in the commercial species European sea bass. A total of 12,158 potential lncRNAs were detected in the head kidney and brain. We found that some lncRNAs were not common for both tissues, and these lncRNAs were located near coding genes that are primarily involved in tissue-specific processes, reflecting a degree of cellular specialisation in the synthesis of lncRNAs. Moreover, lncRNA modulation was analysed in both tissues at 24 and 72 h after infection with nodavirus. Enrichment analysis of the neighbouring coding genes of the modulated lncRNAs revealed many terms related to the immune response and viral infectivity but also related to the stress response. An integrated analysis of the lncRNAs and coding genes showed a strong correlation between the expression of the lncRNAs and their flanking coding genes. Our study represents the first systematic identification of lncRNAs in European sea bass and provides evidence regarding the involvement of these lncRNAs in the response to nodavirus.

## 1. Introduction

European sea bass (*Dicentrarchus labrax* L.) is a very valuable fish species, especially for Mediterranean countries. This species was the first non-salmonid species to be cultured in Europe, and since the 1990s, sea bass aquaculture has grown exponentially and is currently one of the main cultured fish species in Europe [1]. However, different diseases cause important economic losses and represent a major limiting factor for production. These effects are observed for nervous necrosis virus (NNV), or nodavirus, a member of the family Nodaviridae, genus *Betanodavirus*, which can affect numerous aquatic animals, including a wide variety of marine and freshwater fish species [2]. This icosahedral, nonenveloped, positive-sense single-stranded RNA virus is the causative agent of viral encephalopathy and retinopathy (VER), which is characterised by damage to its target tissues, the nervous system (e.g., brain, retina and spinal cord) [3]. Among the four nodavirus genotypes, European sea bass seems to be primarily affected by red-spotted grouper nervous necrosis virus (RGNNV), especially during larval and juvenile stages [2,3].

In recent years, some publications have reported the involvement of numerous immune factors in the defence against nodavirus in different tissues from *D. labrax* [4,5,6,7,8,9,10,11,12]. The development of next-generation sequencing (NGS) technologies also enabled us to analyse the complete transcriptome response after NNV infection both in vitro [13,14] and, more recently, in vivo [15]. Nevertheless, the potential role of noncoding RNAs (ncRNAs) in the modulation of the response to NNV infection in *D. labrax* has not been determined.

The non-coding regions of the genome remained largely unexplored until the last decade. Nevertheless, when it was discovered that the genome is transcribed into many non-coding RNAs (ncRNAs), in addition to the well-known transfer RNAs (tRNAs) or ribosomal RNAs (rRNAs), numerous investigations were conducted in a variety of species in an attempt to identify them and to describe their functions and expression profiles. The long noncoding RNAs (lncRNAs) represent a subset of ncRNAs with a length over 200 nucleotides and transcribed in the same way as mRNA [16]. However, lncRNAs are among least well-understood ncRNAs. This is probably due to the variety of regulatory mechanisms that they could affect [16], but also to their rapid evolution, and consequently, to the absence of recognisable homologs for most of the lncRNAs even in evolutionarily close species [17,18] and to the divergent subcellular location and function of certain conserved lncRNAs [19]. LncRNAs regulate the expression of adjacent genes (*cis*-acting regulation) or genes located at other genomic locations, even on different chromosomes (*trans*-acting) [16]. The gene expression promotion or repression mediated by lncRNAs can be conducted through different mechanisms, including chromatin remodelling, promoter inactivation, transcription interference, activation and/or transport of transcription factors and epigenetic regulation [16]. This wide variety of potential regulatory mechanisms makes it difficult to establish concrete functions for specific lncRNAs. Nevertheless, lncRNAs have been demonstrated to be involved in immune system regulation in mammals [20,21].

In fish, although functional studies remain highly limited, several publications reported the modulation of lncRNAs after different stimuli and/or conditions with a special focus on the immune system [19]. Because lncRNAs can alter the expression of their adjacent genes, the functionality of those lncRNAs identified in fish is usually predicted based on the function of their neighbouring protein-coding genes [22].

In this work, we identified the lncRNA repertoire in the head kidney and brain from European sea bass. We selected these tissues because the head kidney is the main immune and hematopoietic organ in fish and the brain is a target tissue for NNV. Our results showed the existence of tissue-specific lncRNAs, with a specialised role in the maintenance of the basic functions of each tissue, as well as a broad modulation of the lncRNAs after NNV challenge. The functionality of these infection-induced lncRNAs was predicted based on the analysis of their nearby coding genes, revealing a variety of processes in which they could be involved. Because samples were taken at 24 and 72 h post-infection (hpi), we also observed a highly variable lncRNA profile depending on the sampling point, which could be indicative of the fine-tuned gene regulation mediated by these lncRNAs.

## 2. Materials and Methods

### 2.1. Fish and Virus

Juvenile *D. labrax* (average weight 70 g) were obtained from the Estación de Ciencias Mariñas de Toralla (ECIMAT, Universidad de Vigo, Vigo, Galicia, Spain) facilities. Prior to experiments, fish were acclimatised to the laboratory conditions for 2 weeks and maintained in 500 litres fibreglass tanks with a re-circulating saline-water system (total salinity approximately 35 g/L) with a light-dark cycle of 12:12 h at 20–22 °C and fed daily with a commercial diet. Animals were euthanised via MS-222 (Sigma-Aldrich, St. Louis, MO, USA) overdose (500 mg/L). All the experimental procedures were reviewed and approved by the CSIC National Committee on Bioethics under approval number ES360570202001/20/FUN.01/INM06/BNG01.

The nodavirus red-spotted grouper nervous necrosis virus (RGNNV) (strain 475-9/99) was propagated at 25 °C in the SSN-1 cell line (ECACC 96082808) cultured in Leibovitz’s L-15 medium (Gibco, Carlsbad, CA, USA) supplemented with 2 mM glutamine (Gibco), 2% foetal bovine serum (FBS) (Gibco) and 1% penicillin/streptomycin solution (Invitrogen, Waltham, MA, USA). The virus stock was titrated into 96-well plates according to established protocols [23], and aliquots were stored at −80 °C until use.

### 2.2. Fish Infection and Sampling

A total of 18 sea bass were intramuscularly injected with 100 µL of a nodavirus suspension (10^6^ TCID_50_/_mL_), whereas the same number of fish served as the control and were inoculated with the same volume of medium (L-15 + glutamine + 2% FBS + penicillin/streptomycin). Head kidney and brain samples were taken from nine fish at 24 and 72 h post-challenge. The same quantity of tissue from three animals was pooled, obtaining three biological replicates (three fish/replicate) per tissue at each sampling point.

For this challenge experiment, mortality data and viral replication analysis in head kidney and brain from infected fish were previously provided [15].

### 2.3. RNA Isolation and High-Throughput Transcriptome Sequencing

Total RNA from the different samples was extracted using the Maxwell 16 LEV simplyRNA Tissue kit (Promega, Madison, WI, USA) with the automated Maxwell 16 Instrument in accordance with instructions provided by the manufacturer. The quantity and purity of the total RNA was measured in a NanoDrop ND-1000 (NanoDrop Technologies, Inc., Wilmington, DE, USA), and RNA integrity was analysed in the Agilent 2100 Bioanalyzer (Agilent Technologies Inc., Santa Clara, CA, USA) according to the manufacturer’s instructions. All the samples showed an RIN over 8.0; therefore, they were used for Illumina library preparation. Double-stranded cDNA libraries were constructed using the TruSeq RNA Sample Preparation Kit v2 (Illumina, San Diego, CA, USA), and sequencing was performed using Illumina HiSeq™ 4000 technology at Macrogen Inc. Korea (Seoul, Republic of Korea). The read sequences were deposited in the Sequence Read Archive (SRA) (http://www.ncbi.nlm.nih.gov/sra) under the accession number PRJNA589774.

### 2.4. Sequence Assembly and LncRNA Mining

CLC Genomics Workbench, v. 11.0.2 (CLC Bio, Aarhus, Denmark) was used to filter, assemble and perform the RNA-Seq and statistical analyses. Raw reads were trimmed to remove adaptor sequences and low-quality reads (quality score limit 0.05). A reference transcriptome was constructed by de novo assembly using all the samples with an overlap criterion of 70% and a similarity of 0.9 to exclude paralogous sequence variants. The settings used were a mismatch cost = 2, deletion cost = 3, insert cost = 3 and minimum contig length = 200 base pairs. From the de novo assembly, the selection of the lncRNAs was conducted following the pipeline previously described [24] but with some modifications. Briefly, the contigs were annotated using Blastx (e-value < 1 × 10^−5^) against the proteins for all the bony fish species included in the NCBI GenBank and UniProt/Swiss-Prot databases. All the annotated contigs were deleted from the assembly, as well as all the contigs with an average coverage <50. The potential open reading frames (ORFs) were predicted for the remaining contigs, and those with a potential ORF >200 bp were discarded. After this step, the Coding Potential Assessment Tool (CPAT) [25] was used to discard sequences with coding potential. The contigs that passed all the filters were considered putative lncRNAs and retained for further analyses.

### 2.5. Differential Expression Analysis

RNA-Seq analyses of the putative lncRNAs were conducted with the following settings: mismatches = 2, length fraction = 0.8, similarity fraction = 0.8. The expression values were set as transcripts per million (TPM). To identify and quantify the directions of variability in the data, a principal component analysis (PCA) plot was constructed by using the original expression values. Finally, Baggerley’s statistical analysis test was used to compare gene expression levels and to identify differentially expressed lncRNAs. Transcripts with absolute fold change (FC) values >2 and false discovery rate (FDR) <0.05 were retained for further analyses. Those significantly modulated lncRNAs were selected, and the average TMP value of the three biological replicates is represented in heat maps. To this end, the distance metric was calculated by Pearson’s method, and lncRNAs were hierarchically clustered by average linkage. Venn diagrams were constructed with the Venny 2.1 tool (http://bioinfogp.cnb.csic.es/tools/venny/).

### 2.6. Genome Mapping and Identification of LncRNA Neighbouring Coding Genes

To position the putative lncRNAs on the genome, the *D. labrax* genome [26], composed of 25 scaffolds, was uploaded into the CLC Genomics Workbench. These scaffolds were constructed based on linkage/radiation hybrid groups (LG1A - LGx), in which approximately 83% of the contigs were located [26]. The remaining unanchored scaffolds were concatenated into a virtual chromosome (‘UN’) [26].

LncRNAs were mapped using the following parameters: length fraction = 0.8, similarity fraction = 0.8, mismatch cost = 2, insertion cost = 3 and deletion cost = 3. The correlation between the chromosome length and the number of mapped lncRNAs per chromosome was calculated with IBM SPSS Statistics V25.0 by using Pearson’s correlation coefficient (r). The coding genes flanking up to 10 kb upstream and downstream from the mapped and differentially expressed lncRNAs for each comparison were identified and selected for Gene Ontology (GO).

### 2.7. GO Enrichment Analyses

For the significantly differentially expressed lncRNAs in each comparison (Brain 24 h infected vs. Brain 24 h control; Brain 72 h infected vs. Brain 72 h control; HK 24 h infected vs. HK 24 h control; HK 72 h infected vs. HK 72 h control), the lncRNAs-neighbouring coding-genes were extracted for GO enrichment analyses using Blast2GO software [27]. For the GO enrichments, Fisher’s exact test was run with default settings, a p-value cut-off of 0.01 was applied, and the enriched list was reduced to the most specific GO terms. The most enriched biological processes among the protein coding genes proximal to lncRNAs were represented. When the number of significantly enriched GO terms exceeded 30, only the 30 most significant terms were represented.

### 2.8. Correlation Analyses Between LncRNAs and Coding Genes

Correlations in the expression of lncRNAs and their neighbouring genes were calculated by using Pearson’s correlation coefficient and Spearman’s rank correlation coefficient with IBM SPSS Statistics V25.0. Correlations were considered to be significant when p-values were <0.01. To illustrate the expression correlations, heat maps were illustrated with the free software Heatmapper [28] and the average TPM values of the different experimental conditions and sampling points were normalised by row. The protein–protein interaction networks were constructed with String 11.0 (https://string-db.org) [29].

### 2.9. Quantitative PCR (qPCR) Validation of LncRNA Expression

cDNA synthesis of the samples was conducted with the NZY First-Strand cDNA Synthesis kit (NZYTech, Lisbon, Portugal) using 0.5 µg of total RNA. A total of 12 lncRNAs were used to validate the RNA-Seq results. Specific qPCR primers for the selected lncRNAs were designed using Primer 3 software [30], and their amplification efficiency was calculated with the threshold cycle (CT) slope method [31]. Primer sequences are listed in Supplementary Table S1. Individual qPCR reactions were carried out in a 25-µL reaction volume containing 12.5 µL of SYBR GREEN PCR Master Mix (Applied Biosystems, Foster City, CA, USA), 10.5 µL of ultrapure water, 0.5 µL of each specific primer (10 µM) and 1 µL of two-fold diluted cDNA template in MicroAmp optical 96-well reaction plates (Applied Biosystems). Reactions were conducted using technical triplicates in a 7300 Real-Time PCR System thermocycler (Applied Biosystems). qPCR conditions consisted of an initial denaturation step (95 °C, 10 min) followed by 40 cycles of a denaturation step (95 °C, 15 s) and one hybridization-elongation step (60 °C, 1 min). The relative expression level of the lncRNAs was normalised following the Pfaffl method [31] and using *18S ribosomal RNA* (*18S*) as a reference gene.

## 3. Results

### 3.1. Assembly, Annotation and LncRNA Mining

**Figure 1:**
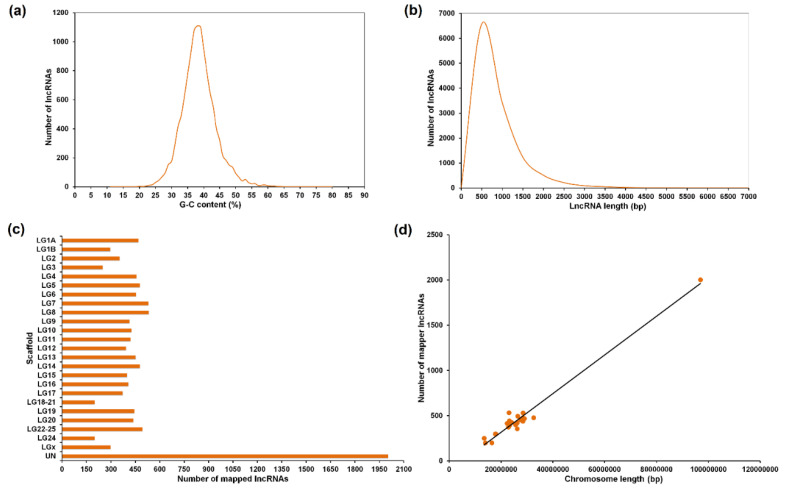
Features of predicted lncRNAs in *D. labrax*. (**a**) Guanine-cytosine (GC) content and (**b**) length distribution of the 12158 predicted lncRNAs. (**c**) LncRNA abundance and localisation per chromosome. (**d**) Correlation between chromosome length and lncRNA abundance.

A summary of the reads, assembly data, contig annotation and lncRNA information is included in Table 1. Approximately 680 million reads were obtained from the different samples of European sea bass with an average of 28 million per sample, and over 99% of raw reads met the quality control standards. From the de novo assembly, a total of 347,317 contigs were obtained with an average length of 723 bp. A total of 69% of the contigs (240,274) were successfully annotated against the bony fish sequences. From the non-annotated contigs (107,043), a total of 12,158 passed all the filters to be considered potential lncRNAs (Supplementary Table S2). The length of these selected contigs ranged from 200 to 6829 bp with an average length of 667 bp (Figure 1a). The GC content (%) of the predicted lncRNAs ranged from 11 to 80% with an average value of 38% (Figure 1b).

From the 12,158 putative lncRNAs, 95.96% (11,667) were successfully mapped to the *D. labrax* genome (Figure 1c). The scaffold with a higher number of lncRNAs was the virtual chromosome ‘UN’ (2004 lncRNAs). This finding is most likely due to the greater length of this virtual chromosome compared to the true ones. Indeed, a strong positive correlation (Pearson’s r = 0.99) existed between the chromosome length and the number of predicted lncRNAs per chromosome (Figure 1d), reflecting that there is no enrichment in lncRNA abundance in any particular chromosome.

### 3.2. Tissue Distribution of the LncRNAs

**Figure 2:**
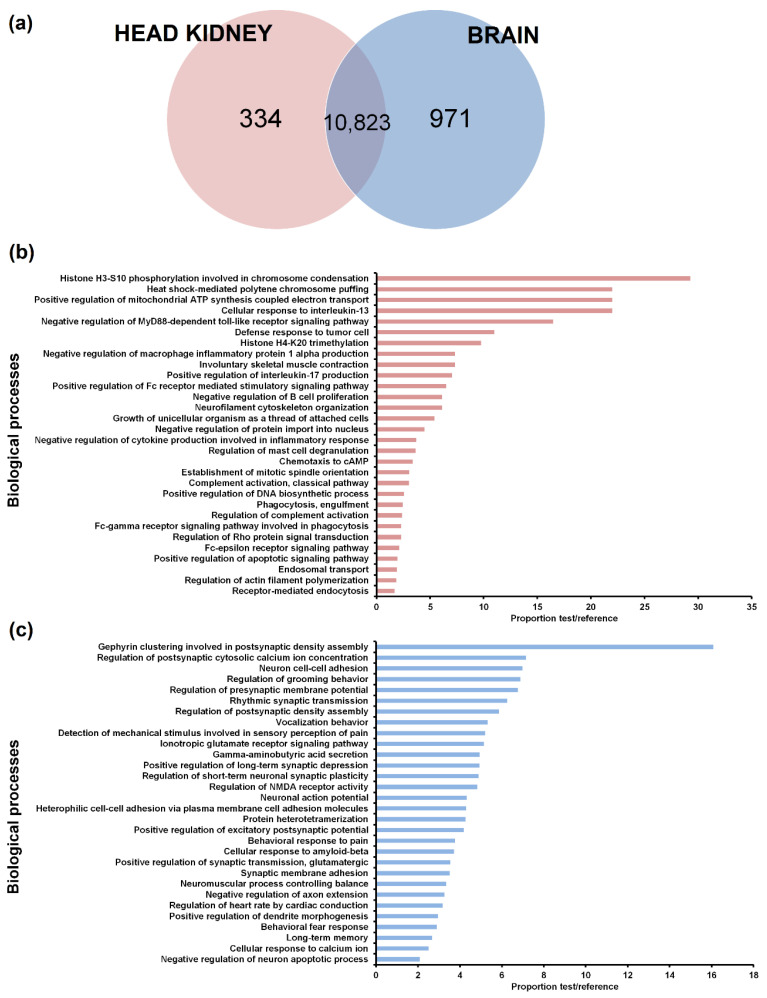
LncRNAs identified per tissue. (**a**) Venn diagram reflecting the number of lncRNAs with a transcripts per million (TPM) value in at least one of the samples from each tissue. Most of the predicted lncRNAs were detected in both the head kidney and brain. In contrast, a total of 30 predicted lncRNAs obtained a TPM value of 0 in all the samples. (**b**,**c**) The neighbouring coding genes of the lncRNAs expressed in the head kidney but not in the brain (**b**) and vice versa (**c**) were analysed by GO enrichment analyses (biological processes). Only the 30 most significant terms were represented.

To identify lncRNAs expressed in head kidney but not in brain and vice versa, only those lncRNAs with a TPM value of 0 in all the samples from the same tissue (12 samples) were considered absent in the corresponding tissue. A Venn diagram of the lncRNAs detected in the head kidney and brain was constructed (Figure 2a). While 30 lncRNAs did not show a TPM value in any of the samples both in head kidney and brain, most of the lncRNAs were detected in both tissues (10823). A total of 971 lncRNAs were expressed in the brain but not in the head kidney, whereas 334 lncRNAs were only found in the head kidney.

As expected, GO enrichment analysis of the lncRNAs expressed only in one of the tissues revealed biological processes directly related to specific aspects of the functionality of each organ (Figure 2b,c). In the head kidney, numerous immune terms were enriched, which were mainly related to the production of cytokines, Toll-like receptor signalling, inflammation, leukocyte activation and proliferation, complement pathway and phagocytosis (Figure 2b). On the other hand, neighbouring genes of those lncRNAs expressed only in the brain showed enrichment in terms directly related to the nervous system (Figure 2c).

To illustrate the relevance of the tissue and of the infection in the lncRNA profile, a PCA score plot was constructed (Figure S1). Interestingly, all the head kidney samples clustered separately from the brain samples, reflecting the higher weight of the tissue compared to the nodavirus infection.

### 3.3. Differential Expression of LncRNAs after Nodavirus Challenge

**Figure 3:**
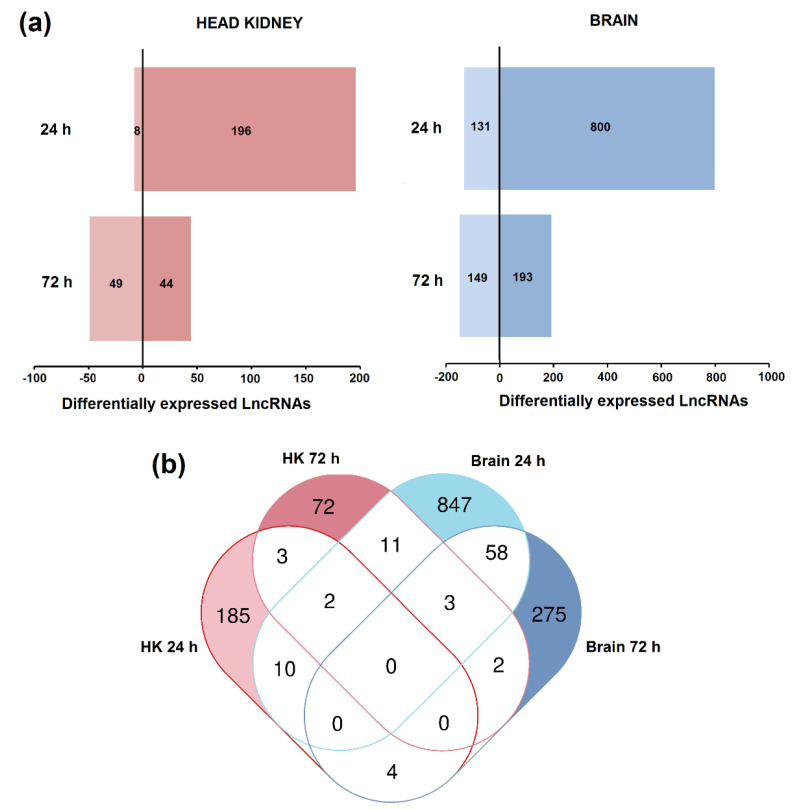
Temporal expression of the predicted lncRNAs after nodavirus infection in head kidney and brain. (**a**) Number of lncRNAs up- and downregulated in each tissue at 24 and 72 hpi with nervous necrosis virus (NNV). (**b**) Venn diagram representing the shared and unshared lncRNAs modulated after NNV challenge in both tissues at the different sampling points.

The differential expression analysis (FC > 2, FDR < 0.05) for each tissue and sampling point is provided in Table S3. A total of 204 and 93 lncRNAs were significantly modulated in the head kidney at 24 and 72 h after nodavirus infection, respectively (Figure 3a). In the brain, 931 and 342 lncRNAs were affected at these sampling points (Figure 3a). In both tissues, the number of upregulated lncRNAs was higher than that of downregulated lncRNAs at 24 h post-challenge, but these differences were substantially reduced after 72 h.

A Venn diagram was constructed to illustrate the shared and unshared lncRNAs modulated in both tissues at different days post-challenge (Figure 3b). Most of the differentially expressed lncRNAs were only modulated in one of the tissues and their expression pattern also completely changed in the same tissue depending on the day after infection (Figure 3b).

**Figure 4:**
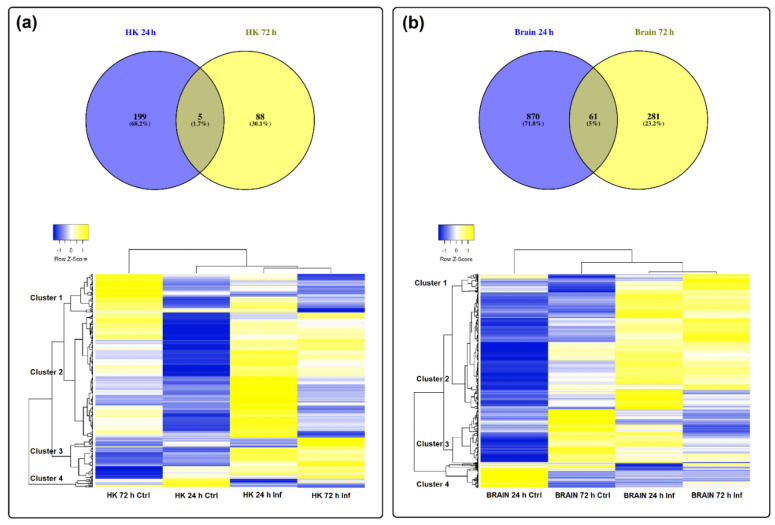
Modulation of lncRNAs in (**a**) head kidney and (**b**) brain after nodavirus challenge. Venn diagrams represent the number of shared and unshared lncRNAs significantly modulated at each sampling point (FC > 2, FDR < 0.05). Heat maps of the lncRNAs significantly affected by the infection in each tissue and hierarchical clustering of the different samples constructed with the TPM values.

Indeed, by analysing the lncRNAs affected by the infection per tissue, we observed that in the head kidney, only 5 lncRNAs were commonly modulated at both 24 and 72 h post-challenge, representing 1.7% of the total lncRNAs affected by the infection in this tissue (Figure 4a). In the brain, the number of shared lncRNAs between both sampling points was 61, representing 5% of the total (Figure 4b). This almost complete switch in the lncRNA profile over time is also reflected in the corresponding heat maps (Figure 4a,b). Interestingly, in both tissues, the pattern of the analysed lncRNAs showed a notably different profile between the control at 24 h and at 72 h (Figure 4a,b). This highlights the importance of including the corresponding controls for each sampling point, especially in the case of a very stressful manipulation for the challenge (anaesthesia and intramuscular injection). However, the absence of a time 0 control did not allow to determine the basal lncRNA expression profile in the analysed tissues, which could be also an interesting question. In general terms, for both HK and the brain, four main clusters of lncRNAs were observed. For HK, cluster 1 included lncRNAs mainly downregulated at 72 hpi, cluster 2 grouped lncRNAs induced at 24 h by nodavirus, cluster 3 included lncRNAs induced at both 24 and 72 h after infection and cluster 4 included lncRNAs downregulated at both sampling points (Figure 4a). In the brain, cluster 1 was mainly composed of lncRNAs overexpressed at 72 h post-challenge, cluster 2 included those lncRNAs induced both at 24 and 72 h, cluster 3 included lncRNAs highly expressed in controls at 72 h and finally, cluster 4 included the lncRNAs highly represented in the control at 24 h (Figure 4b).

### 3.4. GO Enrichment of the LncRNA Neighbouring Coding Genes

**Figure 5:**
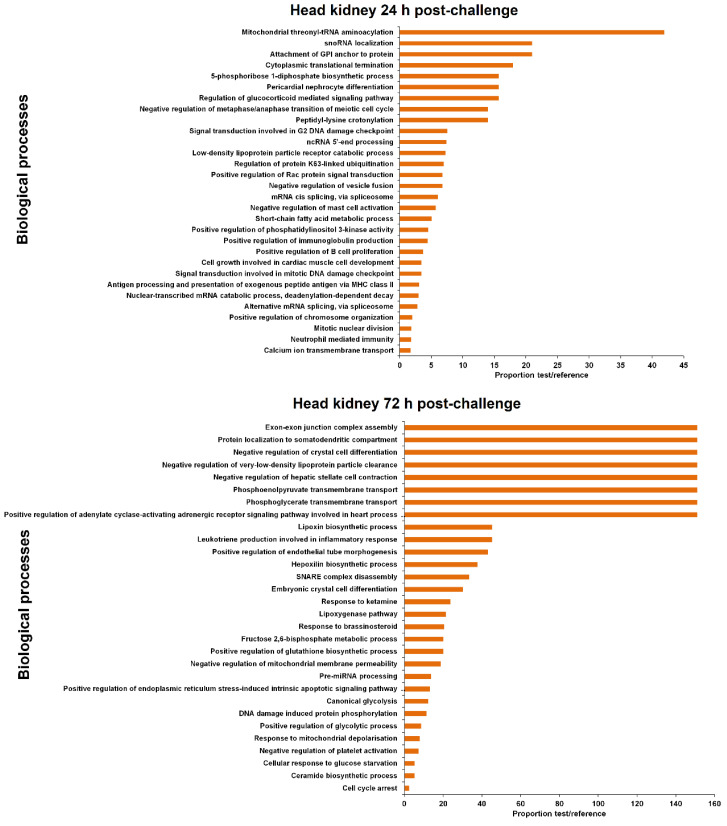
GO enrichment analysis (biological processes) of the neighbouring coding genes of the differentially modulated lncRNAs (FC > 2, FDR < 0.05) in head kidney after viral challenge. Only the 30 most significant terms were represented.

**Figure 6:**
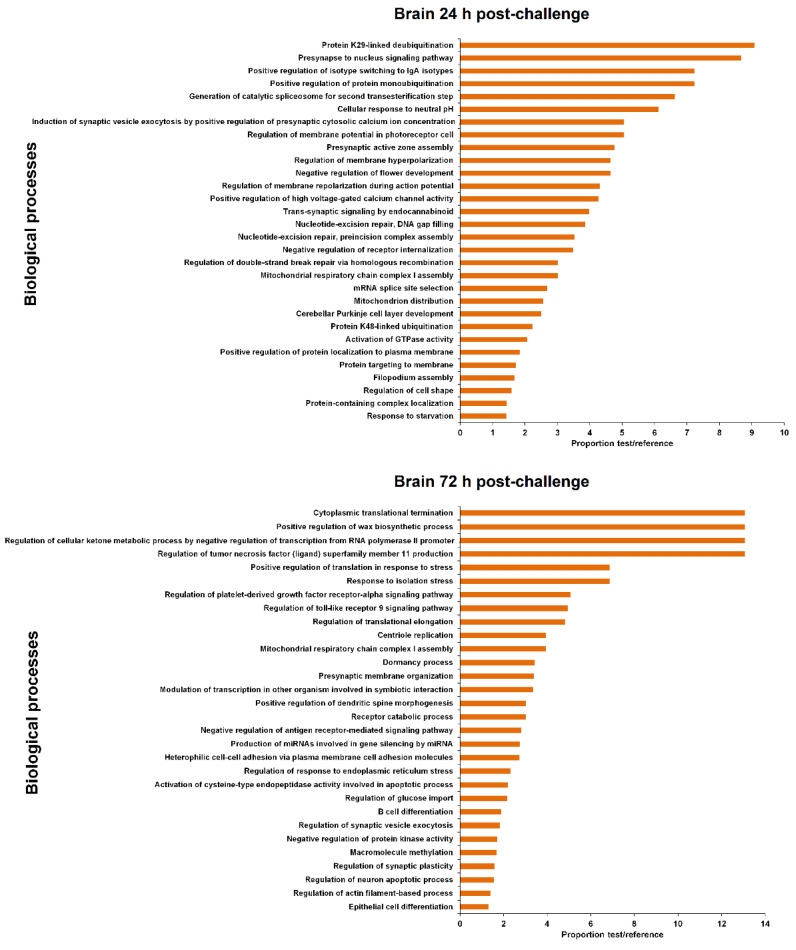
GO enrichment analysis (biological processes) of the neighbouring coding genes of the differentially modulated lncRNAs (FC > 2, FDR < 0.05) in the brain after viral challenge. Only the 30 most significant terms were represented.

The coding genes flanking the differentially expressed lncRNAs were extracted (Supplementary Table S4) for GO enrichment analysis. The 30 most significantly enriched biological processes are represented in Figure 5 and Figure 6 for head kidney and brain samples, respectively.

For head kidney samples (Figure 5), numerous biological process terms directly involved in immunity were found to be enriched at 24 h post-challenge (‘negative regulation of mast cell activation’, ‘positive regulation of immunoglobulin production’, ‘positive regulation of B cell proliferation’, ‘antigen processing and presentation of exogenous peptide antigen via MHC class II’ and ‘neutrophil mediated immunity’). Nevertheless, this immune representation seemed to be diluted at 72 h, although the immune-related term ‘leukotriene production involved in inflammatory response’ also appeared to be enriched. In this tissue, some biological process terms suggesting DNA damage and cell cycle arrest were observed both at 24 h (‘signal transduction involved in G2 DNA damage checkpoint,’ ‘signal transduction involved in mitotic DNA damage checkpoint’) and 72 h post-challenge (‘DNA damage induced protein phosphorylation,’ ‘cell cycle arrest’). At this sampling point, the term ‘positive regulation of endoplasmic reticulum stress-induced intrinsic apoptotic signalling pathway’ was also significantly represented.

In brain samples, the enriched terms were almost completely different from those observed in head kidney (Figure 6). In this case, although a biological process immune term was also found at 24 h (‘positive regulation of isotype switching to IgA isotypes’), the representation of immune processes was lower compared to head kidney. As also occurred in the head kidney, some DNA damage terms were also observed (‘nucleotide-excision repair, DNA gap filling’, ‘nucleotide-excision repair, pre-incision complex assembly’, ‘regulation of double-strand break repair via homologous recombination’), as well as numerous de-ubiquitination-related processes. Moreover, two biological processes related to calcium homeostasis were also represented (‘induction of synaptic vesicle exocytosis by positive regulation of presynaptic cytosolic calcium ion concentration’ and ‘positive regulation of high voltage-gated calcium channel activity’). After 72 h, the representation of immune terms increased in this tissue, with the biological process enriched terms ‘regulation of toll-like receptor 9 signalling pathway’, ‘negative regulation of antigen receptor-mediated signalling pathway’ and ‘B cell differentiation’. Two stress-related biological processes were represented (‘positive regulation of translation in response to stress’ and ‘response to isolation stress’), and as was observed in head kidney, an endoplasmic reticulum (ER) stress term (‘regulation of response to endoplasmic reticulum stress’).

The representation of immune biological processes in both tissues could be directly related to the NNV replication level, since the detection of the virus was higher in the head kidney at 24 hpi compared to the brain, but it remained practically unaltered after 72 h [15]. On the other hand, the NNV detection enormously increased in the brain at 72 hpi [15], which could explain the higher representation of immune-related terms at this sampling point in the brain.

### 3.5. Expression Correlation of LncRNAs and Coding Genes

To compare the magnitude of the modulation after nodavirus infection, we constructed scatter dot plots with the fold-change values of the significantly differentially expressed genes (DEGs) and the lncRNAs in the different samples (FC > 2, FDR > 0.05) (Figure S2). For the DEGs, only those that were successfully annotated were included [15]. In general terms, the fold-change variations of the DEGs were considerably more pronounced compared to those observed for the lncRNAs (Figure S2).

**Figure 7:**
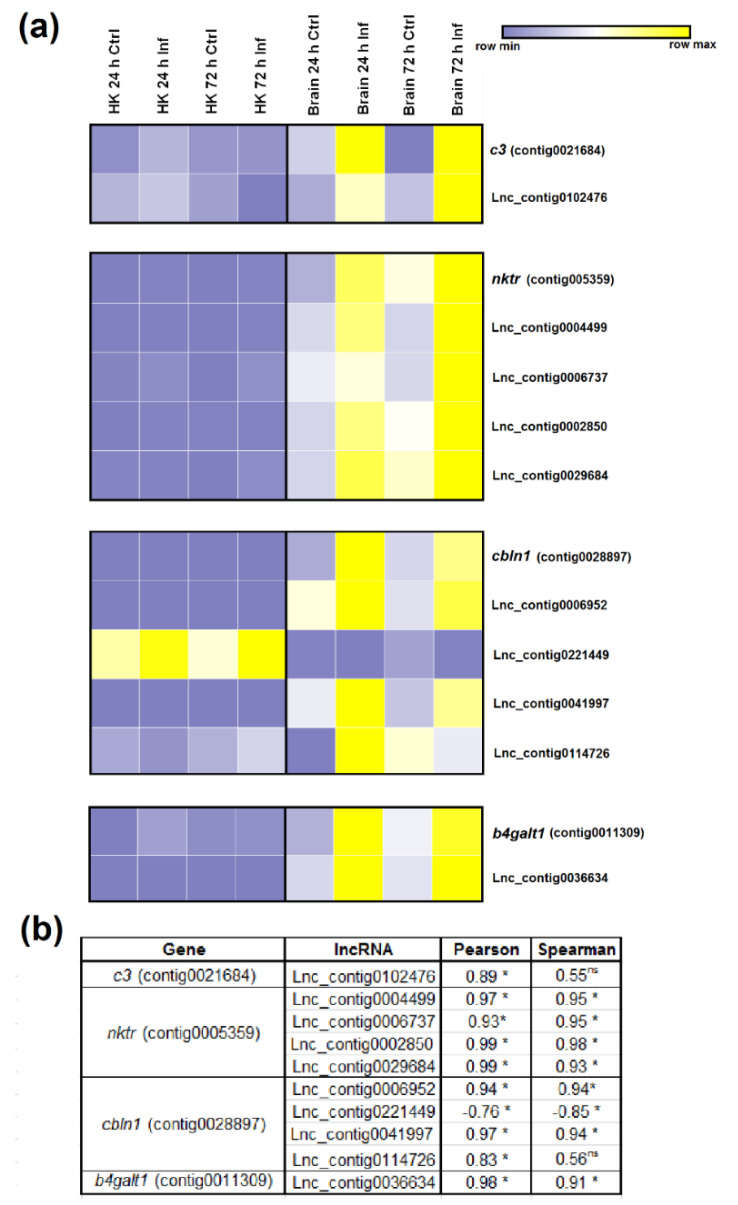
Correlation between differentially modulated coding genes after NNV infection and their flanking lncRNAs. (**a**) Heat map representing the TPM values of four genes and their neighbouring lncRNAs across the different head kidney and brain samples. Expression levels are represented as row-normalised values on a blue–yellow colour scale. (**b**) Correlation values (Pearson’s correlation coefficient and Spearman’s rank correlation coefficient) between the lncRNAs and their nearby coding genes. * represents significant correlation and ns non-significant correlation.

To correlate the expression of the lncRNAs and their flanking coding genes, we randomly selected four DEGs after nodavirus infection [15] with at least one adjacent lncRNA significantly modulated after the viral challenge. The *complement component C3* (*c3*), *NK-tumour recognition protein* (*nktr*), *cerebellin 1* (*cbln1*) and *beta-1,4-galactosyltransferase 1* (*b4galt1*), which were overexpressed in the brain after infection, were represented together with all the predicted lncRNAs flanking and/or overlapping those genes. The TPM values were used to construct the heat maps (Figure 7a) and to calculate the correlation values (Figure 7b). Only one potential lncRNA (Lnc_contig0102476) was found near the *c3* gene, which was significantly upregulated (FC = 3.66) in the brain at 24 hpi. The expression of *c3* and the corresponding lncRNA were strongly correlated, since both were overexpressed in the brain after viral challenge (Figure 7a,b). For *nktr*, four lncRNAs were predicted to be located in the vicinity of the gene (Lnc_contig0004499, Lnc_contig0006737, Lnc_contig0002850, Lnc_contig0029684), and three of them were significantly upregulated in the brain at 24 and/or 72 h after infection. In this case, all the lncRNAs showed the same tendency as that observed for the *nktr* gene (Figure 7a,b). In the case of *cbln1*, four neighbouring lncRNAs were also identified (Lnc_contig0006952, Lnc_contig0221449, Lnc_contig0041997, Lnc_contig0114726), and whereas three of them showed the same pattern as cbln1, the expression of Lnc_contig0221449 was inversely correlated with gene expression (Figure 7a,b). Finally, the lncRNA predicted to be positioned near *b4galt1* (Lnc_contig0036634) was significantly overexpressed in the brain both at 24 and 72 h after NNV infection, and a notably high correlation with gene expression was observed (Figure 7a,b).

### 3.6. LncRNAs Could Mediate Calcium Homeostasis

**Figure 8:**
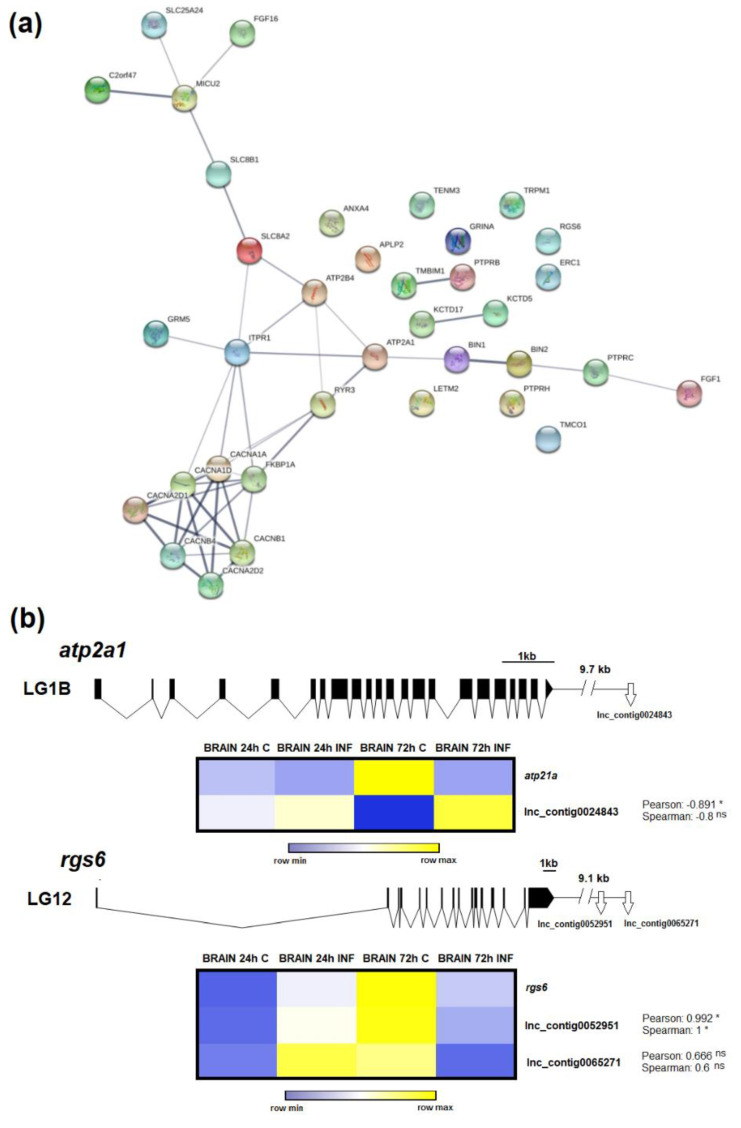
Calcium homeostasis-related genes and their relationship with lncRNAs. (**a**) Protein–protein interaction network of the lncRNAs neighbouring coding genes involved in calcium homeostasis. Numerous lncRNAs significantly modulated in the brain after nodavirus infection were flanked by genes directly related to calcium homeostasis. (**b**) Gene representation of two genes mediating cellular calcium concentration after NNV challenge and genomic location of their neighbouring lncRNAs. Heap maps represent the TPM values of the different contigs. Expression levels are represented as row-normalised values on a blue–yellow colour scale. * represents significant correlation and ns non-significant correlation.

In a previous analysis of the coding transcriptome, we found a strong regulation of genes involved in calcium homeostasis in the brain, and the concentration of this cation seems to be highly altered during NNV infection and is crucial for infectivity [15]. In this work, the biological process terms ‘induction of synaptic vesicle exocytosis by positive regulation of presynaptic cytosolic calcium ion concentration’ and ‘positive regulation of high voltage-gated calcium channel activity’ were also enriched in the brain at 24 hpi (Figure 6), as well as other calcium-related terms not included in the 30 most significantly enriched GO terms but significantly enriched at 24 h and/or at 72 h post-challenge. To illustrate the connections among the different lncRNA neighbouring coding genes involved in calcium homeostasis, a protein–protein network interaction was constructed only for the neighbouring genes of those lncRNAs significantly modulated after viral infection (Figure 8a). Most of the proteins encoded by these genes were interconnected, and the main function of these genes is to directly mediate calcium transport across the cellular, mitochondrial and ER membranes. Among the molecules represented, *sarcoplasmic endoplasmic reticulum calcium atpase 1-like* (*atp2a1* or *serca1*) was the most modulated gene in the coding-transcriptome analysis [15]. On the other hand, the *regulator of g-protein signalling 6-like* (*rgs6*) was slightly modulated [15]. To exemplify the potential involvement of lncRNAs in the modulation of calcium homeostasis-related genes, these two genes were selected, and their expression was correlated to that observed for their neighbouring lncRNAs (Figure 8b). Positive or negative correlations between each coding gene and the corresponding lncRNAs were observed, and these correlations were significant for the *atp21a* gene and the lncRNA Lnc_contig0024843 and for the *rgs6* gene and Lnc_contig0052951. The three lncRNAs were included in the validation of the RNA-Seq results by qPCR.

### 3.7. qPCR Validation of LncRNA Expression

RNA-Seq results were validated by qPCR by analysing the expression of 12 putative lncRNAs significantly modulated in the RNA-Seq results (10 for brain and 2 for head kidney). Although in general terms the magnitude of the modulation was higher in the RNA-Seq data, the qPCR fold-changes followed the same tendency (Figure S3). Indeed, a positive correlation was obtained between the RNA-Seq and qPCR fold-change values by using Pearson’s correlation coefficient (r = 0.84).

## 4. Discussion

In mammals, lncRNAs have been linked to a variety of immune processes, including inflammation [32,33], T cell development, differentiation and migration [34], B cell maturation [35], interferon response [36], dendritic cell differentiation [37] and granulocyte maturation [38]. The lncRNA profile has been shown to be modulated after different viral infections in several species [39,40,41,42]. Moreover, for several lncRNAs, their specific function in viral infection has been elucidated [43].

Massive analysis of zebrafish (*Danio rerio*) lncRNAs enabled the identification of numerous conserved zebrafish lncRNAs with a putative orthologue in mammals [44], which could be indicative of functional conservation. This analysis of the lncRNA repertoire in zebrafish has derived in the creation of a refined database [45], which could help to facilitate the functional analyses in this model species. However, to the best of our knowledge, only one publication has reported the specific immune function of a lncRNA in fish. In that work, the authors found that the PU.1 gene, which is involved in the expression of adaptive immune genes, is negatively regulated by its antisense lncRNA [46]. In the case of zebrafish, the existence of numerous mutant lines could facilitate the identification of lncRNAs related to specific functions. This finding was observed for the heterozygous *rag1*^+/−^ zebrafish, which is partially deficient in the Rag1 protein, an endonuclease involved in the assembly of immunoglobulins and T cell receptor (TCR) genes [47]. When wild-type and *rag1*^+/−^ zebrafish were infected with the spring viremia of carp virus (SVCV), those animals partially deficient in Rag1 showed a modulation of lncRNAs surrounded by genes involved in adaptive immunity, which was not observed in wild-type fish [48]. A plausible explanation is that those animals deficient in adaptive mechanisms potentiated this response to combat the infection compared to the full competent animals [48].

In the past several years, some publications have reported the identification and modulation of lncRNAs in aquacultured fish against a variety of pathogens, especially in salmonids [21,49,50,51,52]. However, to date, no lncRNA analyses have been conducted in European sea bass. We first investigated the modulation of the coding transcripts in head kidney and brain from *D. labrax* infected intramuscularly with nodavirus. In that work, we observed a strong modulation of the stress axis during infection with the virus but a slight regulation of immune-related genes [15].

From the Illumina sequencing of the *D. labrax* transcriptome and de novo assembly [15], we selected those contigs that passed all the filters to be considered potential lncRNAs. We obtained a contig list of 12,158 lncRNAs, and 95.96% of them mapped to the genome. Analysis of the lncRNA abundance per chromosome revealed a homogeneous distribution with a strong positive correlation between the number of putative lncRNAs and chromosome length.

RNA-Seq analysis of these contigs in the different samples showed that some lncRNAs were tissue-specific. This tissue specificity was previously reported for different vertebrate species [53,54], including zebrafish [55] and other teleost species [49]. Most lncRNAs influence the expression of their nearby genes, acting as local regulators; therefore, lncRNA expression is often correlated with the expression of their adjacent coding genes [16]. These tissue-specific lncRNAs were flanked by protein-coding genes involved in biological processes closely linked to the functionality of that tissue. Because the head kidney is a major haematopoietic and immune tissue in fish [56], numerous immune-related GO terms were only enriched in this organ. In the brain, almost all the enriched GO terms were directly related to the maintenance of neuronal functions and homeostasis, neurotransmitters and behaviour.

Nodavirus infection significantly modulated the expression of different lncRNAs in both the head kidney and brain. The brain showed a higher number of modulated lncRNAs (931 and 342 at 24 and 72 h, respectively) compared to the head kidney (204 and 93), probably because of the neurotropic nature of the virus. As was also reported for *S. salar* after infectious salmon anaemia virus (ISAV) infection [49] or *Caligus rogercresseyi* infestation [52], lncRNAs were expressed in a temporal-specific manner with a very low percentage of shared lncRNAs between 24 h and 72 h post-challenge in both tissues. Therefore, the lncRNA transcriptome profile changes as the disease progresses with high spatiotemporal specificity.

GO enrichment analyses of the neighbouring coding genes of lncRNAs modulated after NNV challenge in the head kidney revealed a large number of immune-related terms, especially after 24 h. These terms were mainly related to the activation and proliferation of immune cells, antigen presentation, immunoglobulin production and inflammation. However, the analysis of the transcriptome revealed a practically absent immune response in this tissue, and the modulated genes were mainly related to cortisol synthesis and reactive oxygen species (ROS) production [15]. It is worth mentioning that different DNA damage-, cell cycle- and ER stress-related terms were also enriched in this tissue. It is known that oxidative stress caused by ROS production can induce DNA damage and ER stress, which could lead to cell cycle arrest [57,58,59]. Nevertheless, viruses can manipulate DNA repair pathways and cell cycle control mechanisms to facilitate their own replication [60,61]. On the other hand, ER stress is also intimately related to virus activity. Viruses hijack the host translation machinery to massively produce viral proteins, affecting ER homeostasis and causing ER stress [62]. Moreover, ER stress also induces the production of ROS [63], and ROS are an important component of the immune defence because they can kill pathogens directly by causing oxidative damage or modulating different immune mechanisms [64]. It has been previously described that NNV can induce ROS in European sea bass [15] and oxidative stress-mediated cell death in fish cells [65]. Because ROS are also harmful to the host, the activity of antioxidant molecules, such as glutathione, is also needed to control the damage. The term ‘positive regulation of glutathione biosynthetic process’ was also enriched among those genes flanking the lncRNAs enriched in head kidney at 72 hpi. A previous analysis of the coding genes differentially modulated in sea bass head kidney after NNV infection revealed enrichment in the oxidation-reduction process [15], which could indicate that these are mechanisms controlled by lncRNAs.

In the brain, we found that those genes located at less than 10 kb from differentially modulated lncRNAs after NNV challenge were also enriched in immune terms both at 24 and 72 hpi. At 24 h, only one immune term was enriched in this tissue (‘positive regulation of isotype switching to IgA isotypes’), which was related to antibody production. It has been described in teleosts that host antibody production is an important response to nodavirus infection even at early stages of infection [11,66,67,68], and the expression level of these immunoglobulins could be related to a higher resistance to the virus [69]. Indeed, antibody production is one of the main immune mechanisms protecting the brain against neurotropic viruses [70]. However, in the transcriptome analyses, the production of antibodies seemed to be downregulated at this early sampling point [15]. According to this finding, although more investigations are needed, inhibition of antibody production could be regulated by lncRNAs.

We previously observed that the main response induced by NNV in European sea bass at these early sampling points is the activation of the hypothalamic-pituitary-interrenal (HPI) axis, which is the stress response [15]. According to this study, some stress-related terms were also enriched in the brain for the neighbouring genes of the lncRNAs modulated at 72 hpi. The activation of the stress axis observed in these same samples could lead to calcium influx into the neurons, generating excitotoxicity, and as a consequence, neuronal damage [71,72]. Indeed, calcium cellular homeostasis was found to be highly altered in the brain during NNV infection [15]. For the neighbouring genes of the lncRNAs significantly modulated in this tissue, different calcium-related biological process terms were also enriched, and as occurred in head kidney, we also found lncRNAs flanked by genes related to the DNA damage response, which could be a consequence of excitotoxicity. Although calcium homeostasis modulations in the brain could be a direct consequence of the stress response activated after NNV challenge, viruses can also perturb it and utilise calcium and calcium-related proteins to benefit their own replication [73]. In fact, nodaviruses require the incorporation of calcium ions into the viral capsid for a correct assembly process and integrity [74,75]. We observed that RGNNV, the genotype infecting European sea bass, needs calcium to replicate correctly [15].

Although the number of annotated DEGs in the different tissues at 24 h and 72 hpi was lower compared to the number of differentially modulated lncRNAs, in general terms, the magnitude of these modulations was higher for the DEGs. This observation could suggest that small variations in lncRNA levels can have a high impact on mRNA expression. Moreover, several lncRNAs can simultaneously affect the expression of the same coding gene, and consequently, their effect can be additive. To link the coding-lncRNA response, we randomly selected four genes that were significantly modulated in the brain after viral challenge and flanked by at least one lncRNA that was also significantly affected by the infection. We observed a strong correlation between the expression of the coding genes and all the closely positioned lncRNAs. Because lncRNAs can activate or repress gene expression [16], negative transcriptional correlations can also be observed. Because of the high modulation of calcium homeostasis-related coding genes and lncRNAs closely located to these genes, we suggest that the expression of these genes is likely affected by lncRNAs.

## 5. Conclusions

Taking all these observations into account, we can conclude that there exists a high parallelism between the protein coding genes modulated by NNV challenge and the genes located near lncRNAs affected by infection. Therefore, lncRNAs seem to strongly contribute to the response against nodavirus. Further functional studies between significantly correlated lncRNAs and coding genes will help to elucidate the mechanism of the interactions between lncRNAs and immune genes induced after NNV infection in European sea bass.

## Figures and Tables

**Table 1:** Summary of the Illumina sequencing, assembly, annotation and lncRNA identification.

**READS**	
Total reads	679,746,504
Mean reads per sample	28,322,771
**ASSEMBLY**	
Contigs	347,317
Minimum length	200 bp
Maximum length	26,142 bp
Average length	723 bp
N50	1088 bp
Total assembled reads	215,126,479
**ANNOTATION**	
Annotated contigs	240,274 (69%)
Non-annotated contigs	107,043
**LncRNAs**	
Potential lncRNAs	12,158
Minimum length	200
Maximum length	6829
Average length	667
lncRNAs mapped on genome	11,667 (95.96%)

## Supplementary Materials

The following are available online at https://www.mdpi.com/2079-7737/9/7/165/s1. Table S1: Primer pairs used to validate the lncRNA differential expression analysis, Table S2: LncRNA contig list, Table S3: LncRNA differential expression analyses in head kidney and brain at 24 and 72 h post-challenge with NNV, Table S4: Predicted neighbouring coding genes of the potential sea bass lncRNAs, Figure S1: Principal component analysis (PCA) of the lncRNAs in the different samples, Figure S2: Magnitude of transcriptome modulation in the head kidney and brain after NNV infection, Figure S3: Validation of differentially expressed lncRNAs through qPCR.
