# Supplementary material for: Potential Involvement of lncRNAs in the Modulation of the Transcriptome Response to Nodavirus Challenge in European Sea Bass (Dicentrarchus labrax L.)
Source: Biology (Basel). 2020 Jul 15;9(7):165. doi: 10.3390/biology9070165 (PMC7407339; doi:10.3390/biology9070165)

Supplementary Figure S1. Principal component analysis (PCA) of the lncRNAs in the different samples. Each point represents a biological replicate, and different colours represent the different tissues. Samples were mainly clustered according to the tissue (head kidney or brain) with independence of the NNV infection.

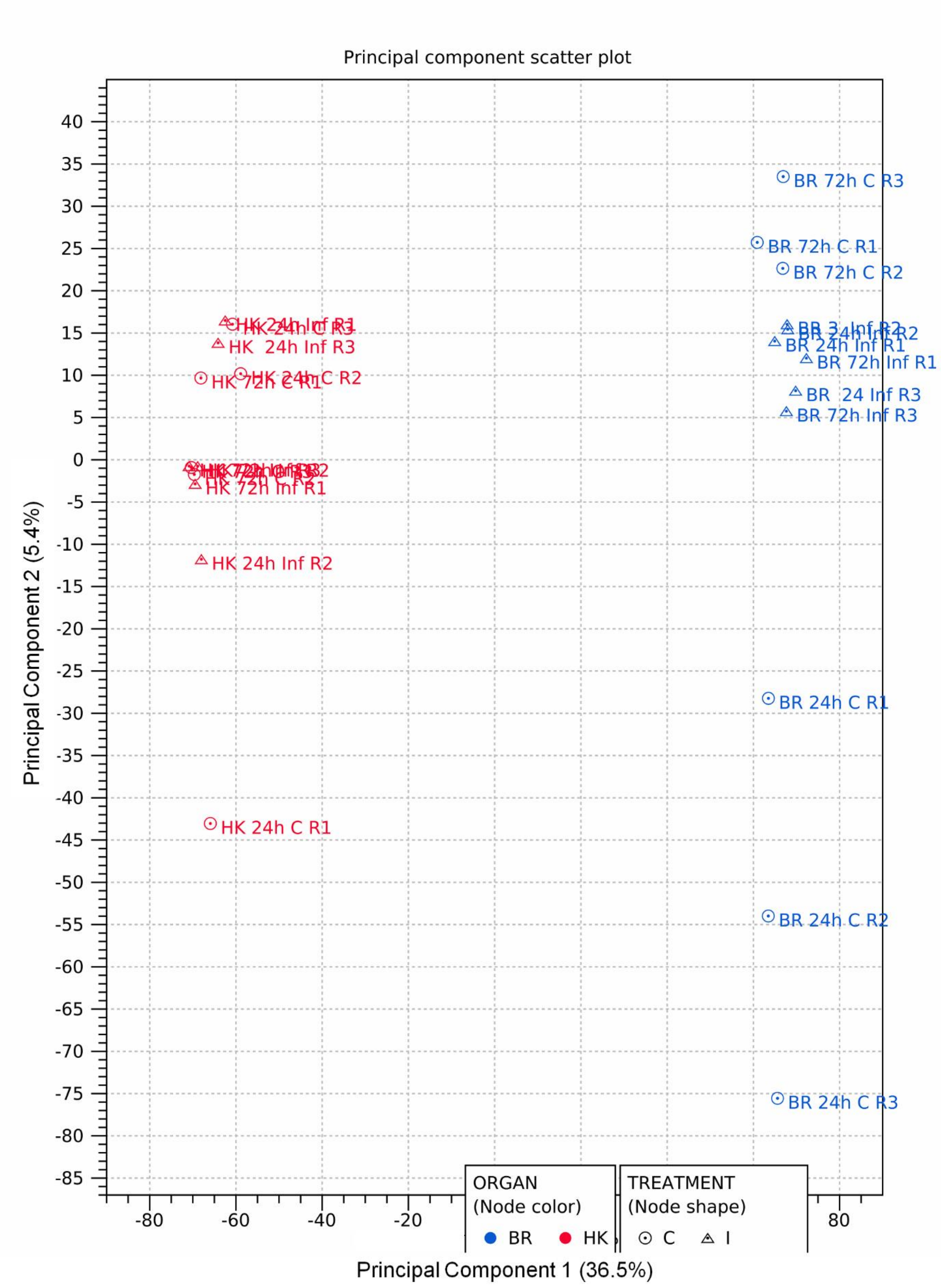

Supplementary Figure S2. Magnitude of transcriptome modulation in the head kidney and brain after NNV infection. (a) Fold-change values (FC>2, FDR<0.05) of the differentially expressed coding genes. **(b)** Fold-change values of the differentially expressed lncRNAs (FC>2, FDR<0.05). Graphs represent the individual FC values, the mean and the range.

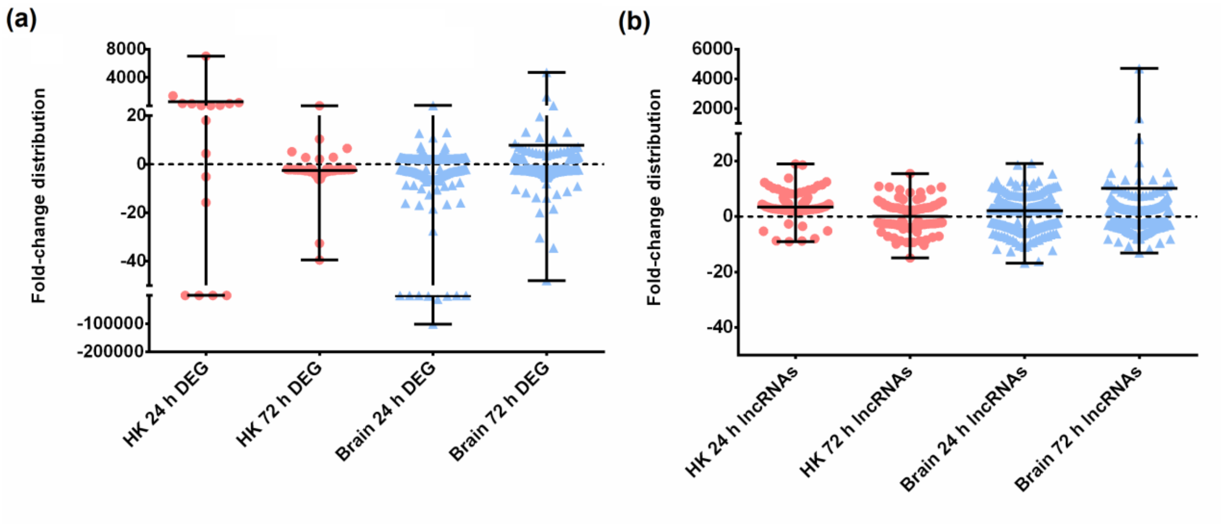

Supplementary Figure S3. Validation of differentially expressed lncRNAs through qPCR. Twelve potential lncRNAs were validated. Pearson's correlation test showed a value of 0.84.

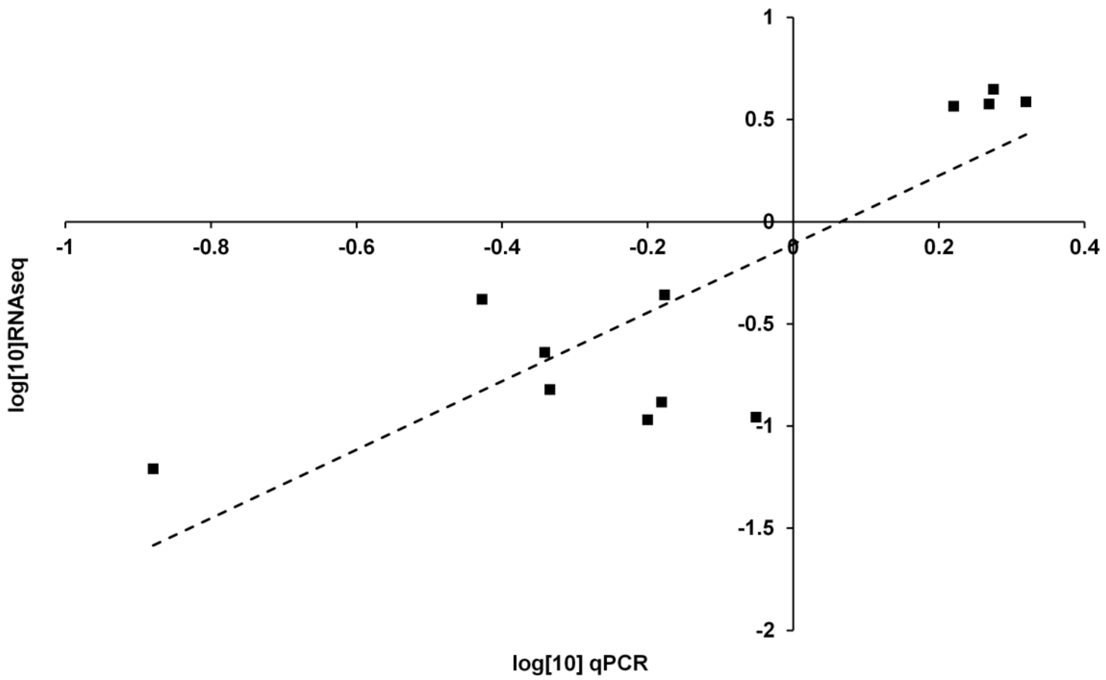

Supplement: Supplementary file 1 [file biology-09-00165-s001.zip › Supplementary Material/Supplementary Figures S1-S3.pdf]
